# Lectures on Nervous Diseases

**Published:** 1890-03

**Authors:** 


*Lectures on Nervous Diseases.* By AMBROSE L. RANNEY, A.M., M.D. Philadelphia: F. A. DAVIS. 1888.

Of the seven sections of which Dr. Ranney's handsome volume is composed, the first is on the anatomy and physiology of the cerebro-spinal axis, and is illustrated with numerous explanatory diagrams, which, if they *are* very diagrammatic and many also vividly coloured, are helpful to the quick and easy apprehension of the statements in the text; they serve also to show that Dr. Ranney's views on the localisation of function are not always identical with those commonly held in this country. Section two is devoted to the detailed instruction how to examine the nervous system of a patient and contains much useful information, never perhaps so conveniently collected together before. The third and fourth sections treat of the individual diseases of the brain and spinal cord fairly well; but inadequately in proportion to the other departments of the work. Section five will to neurologists in this country present most of novelty in its statements; it treats of "functional" nervous diseases, such as epilepsy, chorea, neurasthenia and migraine, and sets forth in a wonderful manner the author's belief that most cases of these diseases are dependent upon anomalies of the ocular muscles and of eye-strain, a belief which is probably shared in its entirety with the author by no one besides Dr. G. T. Stevens, his preceptor in this faith. Nearly three years ago, the New York Neurological Society appointed a commission to investigate the relation between the ocular muscles and "functional" nervous diseases. The report of this commission has recently been presented to the Society, and is strongly adverse to Dr. G. T. Stevens' "treatment of ocular irritations as a therapeutic measure in certain neuroses." The last section, on Electricity in Medicine, consists very largely of a reproduction of his previously published work on that subject, which is here treated in considerable detail and minuteness. We presume Dr. Ranney finds electricity an agent of more remedial power than physicians on this side of the Atlantic habitually do. Static electricity he uses very extensively with elaborate apparatus and apparently with encouraging results.

If, upon the whole, we cannot recommend Dr. Ranney's book as a guide of unexceptionable trustworthiness to the beginner, there is very much in it of high value to the fairly-trained neurologist.

